# Obtaining accurate glucose measurements from wild animals under field conditions: comparing a hand held glucometer with a standard laboratory technique in grey seals

**DOI:** 10.1093/conphys/cox013

**Published:** 2017-02-27

**Authors:** Kimberley A. Bennett, Lucy M. Turner, Sebastian Millward, Simon E. W. Moss, Ailsa J. Hall

**Affiliations:** 1 Division of Science, School of Science, Engineering and Technology, Abertay University, DundeeDD1 1HG, UK; 2 Marine Biology and Ecology Research Centre, Plymouth University, Drake Circus, PlymouthPL4 8AA, UK; 3 NERC Sea Mammal Research Unit, Scottish Oceans Institute, University of St Andrews, St Andrews, FifeKY16 8LB, UK

**Keywords:** Glucose, glucometer, phocid, pinniped, point-of-care, validation

## Abstract

Glucose is an important metabolic fuel and circulating levels are tightly regulated in most mammals, but can drop when body fuel reserves become critically low. Glucose is mobilized rapidly from liver and muscle during stress in response to increased circulating cortisol. Blood glucose levels can thus be of value in conservation as an indicator of nutritional status and may be a useful, rapid assessment marker for acute or chronic stress. However, seals show unusual glucose regulation: circulating levels are high and insulin sensitivity is limited. Accurate blood glucose measurement is therefore vital to enable meaningful health and physiological assessments in captive, wild or rehabilitated seals and to explore its utility as a marker of conservation relevance in these animals. Point-of-care devices are simple, portable, relatively cheap and use less blood compared with traditional sampling approaches, making them useful in conservation-related monitoring. We investigated the accuracy of a hand-held glucometer for ‘instant’ field measurement of blood glucose, compared with blood drawing followed by laboratory testing, in wild grey seals (*Halichoerus grypus*), a species used as an indicator for Good Environmental Status in European waters. The glucometer showed high precision, but low accuracy, relative to laboratory measurements, and was least accurate at extreme values. It did not provide a reliable alternative to plasma analysis. Poor correlation between methods may be due to suboptimal field conditions, greater and more variable haematocrit, faster erythrocyte settling rate and/or lipaemia in seals. Glucometers must therefore be rigorously tested before use in new species and demographic groups. Sampling, processing and glucose determination methods have major implications for conclusions regarding glucose regulation, and health assessment in seals generally, which is important in species of conservation concern and in development of circulating glucose as a marker of stress or nutritional state for use in management and monitoring.

## Teaser

Hand-held point-of-care devices are increasingly used in wildlife settings because they are simple, portable, cheap and use small amounts of blood. However, our data show low accuracy of a glucose meter across different ages and physiological states in grey seals. Glucometers need rigorous testing before use in wildlife species.

## Introduction

Development of rapid, field accessible and informative markers of stress and nutritional state are useful for conservation efforts in vertebrate species. Glucose is a vital metabolic fuel for glycolytic tissues (Winkler *et al*., 2003; Grundy, 2004; Mergenthaler *et al*., 2013). In most mammals, glucose is tightly regulated through modulation of endogenous glucose production (EGP), and glucose uptake and oxidation. Significant deviations in blood glucose levels can cause serious acute and chronic metabolic disturbance and cell death (e.g. Cryer *et al*., 2003; Kawahito *et al*., 2009). Rapid glucose mobilization is essential for acute stress responses, which confer a survival advantage. In vertebrates, chronically elevated glucose levels may result from persistent, repeated stress or cortisol exposure (Barton *et al*., 1987; Khani and Tayek, 2001), whereas fasting and depletion of body energy reserves can lower circulating glucose (Cherel *et al*., 1988). Glucose levels correlate with habitat quality, reproductive state and/or body condition in many bird species (Fairbrother *et al*., 1990; Ruiz *et al*., 2002; Lill, 2011; Minias and Kaczmarek, 2013; Kaliński *et al*., 2014), and can therefore be useful as an indicator of stress, condition or nutritional status in a conservation context.

Stable or increasing seal populations in UK waters is one of the indicators of Good Environmental Status (GES) in the European Marine Framework Strategy Directive (MFSD). Grey seals (*Halichoerus grypus*) are the most abundant phocids in UK waters (SCOS, 2015). Ensuring the effectiveness of MFSD policy requires knowledge of the drivers of grey seal population change, which include stress and energy balance (Hall *et al*., 2001, 2002). Glucose levels may be useful as physiological ecology markers for conservation efforts in seals globally if blood glucose can be measured more rapidly and cheaply than other stress markers, such as glucocorticoids. Seals typically have high postprandial and fasting glucose levels and EGP compared with similar sized terrestrial animals (Schweigert, 1993; Hall, 1998; Keith and Ortiz, 2006; Houser *et al*., 2012, 2013; Schermerhorn, 2013), which can make interpretation of their glucose measurements challenging.

Whilst determination of glucose levels from processed plasma is considered the gold standard (Haanestad and Lundblad, 1997), the ability to measure blood glucose in real time in wild seals is of great appeal because it would reduce volumes of blood taken, remove the need for centrifugation (Haeckel *et al*., 2002), abrogate processing delays that result in glycolysis (Lin *et al*., 1976; Chan *et al*., 1989; Fobker, 2014) and avoid addition of preservatives that interfere with enzymes used in glucose determination (Waring *et al*., 2007; Tonyushkina and Nichols, 2009). Additives can also render the remaining sample useless for determination of other analytes, which is problematic when sample number or volume is limited for ethical or logistical reasons.

Glucometers are a vital point-of-care (POC) technology used in humans to manage conditions that result in hyper- or hypoglycaemia (Tonyushkina and Nichols, 2009). Glucometer performance relative to laboratory clinical analysis has been tested for humans (Frishman *et al*., 1992; Brunner *et al*., 1998) and companion animals (Cohn *et al*., 2000; Weiss and Reusch, 2000; Cohen *et al*., 2009; Johnson *et al*., 2009; Summa *et al*., 2014). POCs designed for humans have been used in field conditions for measurements in wildlife species (reviewed by Stoot *et al*., 2014). However, it is important to define their technical accuracy against standard laboratory methods (Lindholm and Altimiras, 2016), a practice that has often been overlooked in non-domesticated animals. Validations of glucometers in wild mammals are scarce (Stoot *et al*., 2014).

We sampled wild, healthy grey seals to determine (i) whether field glucometer readings correlate with plasma measurements in matched samples to assess the performance of the device for future work; (ii) the effect of time delays on sampling and processing of the blood samples on laboratory determination of glucose and (iii) effects of age, physiological and development state and time of day on glucose measurements, which can inform evaluation and interpretation of normal glucose values and appropriate sampling regimes for health assessment, management and conservation efforts.

## Materials and methods

### Animal handling and sampling protocol

Grey seals were sampled on the Isle of May breeding site, Scotland (56° 12’ N, 2° 32’ W) during October–December 2012. Capture and handling complied with the Animal (Scientific Procedures) Act 1986 and were performed by licenced personnel under Home Office Licence 60/4009. Adult females were observed daily throughout lactation, from when they were first identified from either brands or flipper tags. Pup date of birth was recorded where possible. Mother–pup pairs (*n* = 20) were sampled early (Day 5) and late (Day 15) in the suckling period. Pups were assumed to have weaned when the female was not seen in attendance for a full day (Bennett *et al*., 2007, 2015). After weaning, pups were located on the colony and sampled again early (Day 5 postweaning; *n* = 17) and late (Day 15 postweaning; *n* = 13) in the fasting period (Bennett *et al*., 2015).

Each time they were captured, adult females were anaesthetized using a 1 ml 100 kg^−1^ intramuscular dose of Zoletil_100_^TM^ (Virbac, Cedex, France) delivered using a pressurized dart. Length, axillary girth and mass of mothers and pups were recorded as previously described (Pomeroy *et al*., 1999; Bennett *et al*., 2007). On first capture, pups were tagged in the interdigital webbing (Rototag; Dalton ID Systems, Henley on Thames, Oxon, UK) to allow subsequent identification (Fedak and Anderson, 1982). A plasma sample and glucometer readings were taken at each capture. Blood was drawn from the epidural sinus using either 19 gauge, 2 inch needles or 18 gauge 3.5 inch spinal needles, whichever was most appropriate for animal size, to fill a sterile 10 ml potassium ethylenediaminetetraacetic acid (K_3_ EDTA) Vacutainer (Becton Dickinson, Oxon, UK) as previously described (Bennett *et al*., 2015). Tubes were kept on cold packs until processing. After the final draw, and within 2 min of the blood sample collection, one glucometer strip (One Touch^®^ Ultra^®^; Lifescan, Miliptas, CA) was removed from the sealed packet. A spot of the venous blood from the needle was transferred to the reaction site and the strip placed immediately into the glucometer (One Touch^®^ Ultra^®^) according to the manufacturers’ instructions. This was repeated once more to determine precision. Time of sample draw and glucometer reading were recorded in each case.

### Blood processing

Vacutainers were processed immediately on return to the laboratory by centrifugation at 2000 *g* for 15 min. The plasma portion was drawn from the clot using a glass Pasteur pipette and frozen in 500 μl aliquots at −20°C until processing. Time taken to remove plasma from the clot and to freeze it were recorded.

### Glucose measurement

Plasma samples (200 μl) were deproteinized, centrifuged and assayed for glucose in a microplate format using a VERSAmax^TM^ plate reader (Molecular Devices, 73 Sunnyvale, CA, USA) at 410 nm using the glucose oxidase method according to Webster (1996). Visibly haemolysed samples were not analysed.

### Statistical analysis

Statistical analyses were performed in RStudio (Version 0.99.893—© 2009–2016; RStudio Team, 2015). We investigated the relationship between the glucose estimates from the glucometer and the laboratory measurement of plasma within each age/developmental state (i.e. mother or pup, and early or late in suckling or postweaning fast) of the animals using Pearson's correlation. Linear mixed effect models (LMEs) were used to investigate whether the discrepancy between plasma glucose and glucometer reading could be explained by plasma glucose concentration, when in the season the sample was taken (an index of operator experience), and time elapsed between sampling and removing plasma (to establish the effect of sample processing time). An LME was then performed to investigate the effect of developmental stage (mother or pup), physiological state (early or late in suckling, and early or late in fasting), time of day and, where appropriate, when in the season the sample was taken, time from sampling to removal from clot and time from removal from clot to freezing on glucose measurements from both the glucometer and laboratory method. Since time of day is a circular value (Fisher, 1993; Bennett *et al*., 2001), local time was transformed to both cosine and sine values (cosToD = cos(*h*/23) × 360 and sinToD = sin(*h*/23) × 360, respectively). Developmental stage, physiological state, time elapsed between sampling and removing the plasma from the cellular portion, and the time taken between removal from the cellular portion and freezing (Clark *et al*., 1990; Morris *et al*., 2002) and cosToD and sinToD were included as fixed effects. All LMEs were fitted using maximum likelihood and included individual as a random effect to control for repeated measures. Model selection was performed using forward stepwise regression and models were compared using the ‘anova’ function in R (Bennett *et al*., 2015).

## Results

### Performance of glucometer relative to a laboratory method

**Figure 1:**
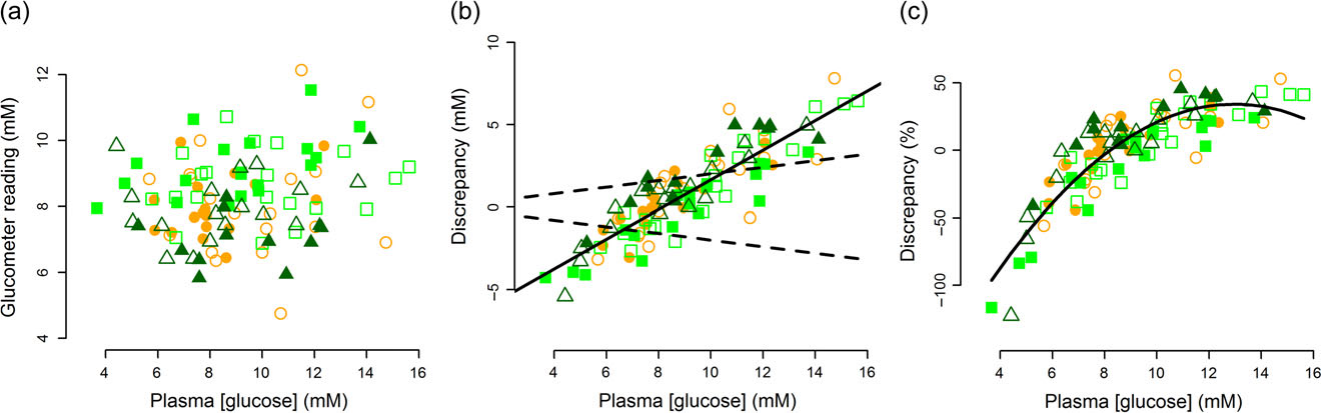
Relationship between plasma glucose concentration measured in the laboratory using the glucose oxidase method and (**a**) the glucometer venous blood glucose reading, (**b**) absolute discrepancy and (**c**) percentage discrepancy of the glucometer venous blood glucose reading. Plasma samples were obtained at the same time as venous glucometer readings from adult female grey seals (circles) during early (Day 5; *n* = 19; open symbol) and late lactation (Day 15; *n* = 17; closed symbol); their pups (squares) during early (*n* = 20; open symbol) and late suckling (*n* = 19; closed symbol); and the same pups (triangles) early (Day 5; *n* = 17; open symbol) and late (Day 15; *n* = 13; closed symbol) in the postweaning fast. Solid trendlines drawn for illustrative purposes. In (b) dashed lines represent 20% glucometer accuracy across the concentration range, which is deemed acceptable for clinical purposes in humans.

Characteristics of the mother pup pairs, glucometer readings and corresponding laboratory values and their correlations are shown in Table 1. There was a weak, positive correlation between plasma glucose and glucometer readings in pups late in the suckling period (Fig. 1a). No correlations existed between the two methods in the other age or developmental/physiological state categories. The co-efficient of variation for the glucometer was 4.83 ± 4.76%. There was no significant relationship between when in the season the sample was taken and precision of the glucometer measurement (LME: *T* = 0.32; *P* = 0.751; df = 57; Akaike's Information Criterion (AIC) = 632.83; *n* (obs) = 105; *n* (individuals) = 43).

**Table 1:** Mean ( ± sd) mass; length; girth; glucose measured immediately in the field using a hand held glucometer (One Touch® Ultra®) (glucometer reading); and glucose levels in plasma (Plasma glucose) of grey seal mothers and pups early and late in the suckling period and pups early and late in the fast after weaning

	Adult females		Pups			
	Early suckling	Late suckling	Early suckling	Late suckling	Early fast	Late fast
Mass (kg)	169.07 (16.72)	135.11 (11.18)	25.51 (3.21)	41.97 (4.86)	40.79 (3.80)	37.62 (4.29)
Length (cm)	175.63 (4.95)	170 (5.35)	95.92 (3.45)	104.05 (4.66)	102.65 (4.50)	102.08 (5.99)
Girth (cm)	146.26 (13.27)	132.29 (9.94)	77.69 (4.52)	97.89 (6.26)	96.00 (4.72)	91.92 (3.95)
Glucometer reading (mM)	8.15^a,d^ (1.75)	8.01^a,c,e^ (0.95)	8.62^b,d,e^ (0.95)	9.21^b^ (0.96)	7.96^a,c^ (0.96)	7.25^c^ (1.09)
Plasma glucose (mM)	9.69^a,c^ (2.51)	8.21^b^ (1.82)	9.74^a,c^ (2.4)	8.95^a,b,c^ (2.69)	8.34^a,b,c^ (2.51)	9.89^c^ (2.56)
Correlation	0.17	0.36	0.05	**0.51**	0.15	0.43
*T*	0.48	1.47	0.21	**2.41**	0.57	1.57
df	17	15	18	**17**	15	11
*P*	0.636	0.161	0.840	**0.027**	0.576	0.145
Difference	1.54^a^ (2.89)	0.19^a^ (1.73)	1.12^a,b^ (2.73)	−0.25^b^ (2.36)	0.38^a^ (2.55)	2.34^c^ (2.32)
Sampling–separation (min)	89.74^a,b,c^ (24.49)	101.25^a^ (29.05)	89.25^a,b^ (23.57)	102.05^a^ (28.13)	74.65^b,c^ (18.33)	72.46^c^ (20.74)
Separation–freezing (min)	81.84^a,b^ (116.56)	75.35^a^ (52.75)	46.15^b^ (58.99)	73.58^a^ (51.01)	18.12^c^ (16.78)	20.08^b,c^ (5.93)

Correlation between the two glucose measurements, *T*, degrees of freedom and *P*, the mean absolute difference between the two glucose measures, time elapsed between sample collection and removal from the cellular portion; and time taken from separation to freezing. Bold font indicates a significant positive correlation between the glucometer and plasma glucose readings (Pearson's correlation co-efficient). Within the glucometer, plasma glucose, difference, time between sampling and separation, and time between separation and freezing rows, cells that share at least one same letter superscript are not significantly different from each other (LME: *P* < 0.05).

The range of accuracy of the glucometer relative to our plasma measurement was 0.5–122%. Absolute and percentage discrepancy of the venous glucometer reading relative to the plasma glucose concentration is shown in Fig. 1b and c. There was a significant positive relationship between the plasma glucose concentration and the absolute discrepancy between the laboratory and glucometer measurements (Plasma-glucometer: LME: *T* = 20.26; *P* < 0.0001; df = 56; AIC = 334.29; *n* (obs) = 105; *n* (individuals) = 43), such that the glucometer overestimates values below ~ 8 mM and underestimates at values above 8 mM. The percentage inaccuracy was much greater at lower measured plasma glucose values. The degree of accuracy of the glucometer compared with the plasma measurement differed between age/stage categories. The glucometer discrepancy compared with laboratory measurements was smallest in the late suckling pups, and tended to overestimate values in these animals. The discrepancy was greatest and the glucometer tended to overestimate glucose in late weaned pups. The discrepancy between the glucometer reading and laboratory measurement was similar between adults early and late in lactation, pups early in lactation and pups early in the postweaning fast. The glucometer underestimated the true plasma value in these age categories (Table 1; Fig. 1b and c).

### Effects of sample processing, age stage and time of day on glucose measurements

Plasma glucose levels were best explained by the developmental/physiological state of the animal and the time elapsed between sampling and removal of plasma from the cellular portion of the blood. Samples from weaned pups were processed significantly faster than those from pups and females late in suckling (Table 1; LME: df = 57; *n* (obs) = 105; *n* (individuals) = 43). However, developmental/physiological differences in laboratory measured glucose were not an artefact of processing time because the model specifically accounted for effects of time elapsed from sampling to removal from clot, and weaned pups did not have the lowest glucose levels. There was a significant decrease (by 1.77 ± 0.79 mM) in plasma glucose in adult females from early (9.69 ± 2.51 mM) to late (8.21 ± 1.82 mM) lactation (LME: *T* = 2.17; *P* = 0.034; df = 56; AIC = 498.92; *n* (obs) = 105; *n* (individuals) = 43). The late lactation values for females were also lower than those in their pups early in the suckling period (LME: *T* = 2.56; *P* = 0.013; df = 56) and late in the postweaning fast (LME: *T* = 2.19; *P* = 0.033; df = 56). There were no other differences in glucose levels between the different developmental/physiological states.

Given the differences in plasma glucose measured in the laboratory between developmental/physiological states described above, there was a small increase in measured plasma glucose with time elapsed between sampling and separation of the plasma from the clot across all developmental/physiological states (LME: glucose = 0.024 (time elapsed) + 5.81; *T* = 2.34; *P* = 0.022; df = 56). Time taken from removal from clot to freezing did not account for any additional variation in plasma glucose levels (ANOVA: *L* ratio = 0.130; *P* = 0.288). Glucose levels were not related to time of day (sinToD: LME: *T* = 0.15; *P* = 0.880; cosToD: LME: *T* = 0.09; *P* = 0.282). Inclusion of time of day with age/stage category and time elapsed between sampling and separation of the plasma from the clot did not improve the model fit (sinTOD: ANOVA: *L* ratio = 0.145; *P* = 0.699; cosTOD: ANOVA: *L* ratio = 1.02; *P* = 0.314). In addition, the time elapsed between sampling and removal from the clot did not contribute to the discrepancy between the glucometer and plasma measurements (LME: *T* = 1.03; *P* = 0.307; df = 55; AIC = 506.57; *n* (obs) = 105; *n* (individuals) = 43).

## Discussion

### Performance of glucometer relative to a laboratory method

We chose the One Touch^®^ Ultra^®^ (Lifescan, Miliptas, CA) device because it was widely available at the time of the study, affordable, can tolerate a wide range of haematocrit (Hct), temperature and humidity and performs well in humans with hyperglycaemia (Brunner *et al*., 1998), which is similar to the normal blood glucose range for grey seals (Bennett *et al*., 2013; Hall, 1998; Schweigert, 1993). Glucometer precision was extremely high. The device was easy to use and gave reproducible readings, even when the operator was inexpert. This is a clear benefit when field teams change or include inexperienced volunteers. However, glucometer accuracy was very poor relative to the laboratory method. The American Diabetes Association recommends readings from glucometers should be within 15% of laboratory-derived values to be usable (Stahl *et al*., 2001). Many organizations allow an accuracy of 20%. Only 32% of our glucometer readings were within 15% of the laboratory glucose measurement and less than half (47%) fell within 20% accuracy. Accuracy was highest between 8 and 10 mM, which is the typical range of blood glucose measurements in grey seals (Schweigert, 1993; Hall, 1998; Bennett *et al*., 2013). In dogs, some glucometers show high levels of inaccuracy that are concentration dependent, similar to our findings (Cohen *et al*., 2009). In elephant seals (*Mirounga angustirostris*), the iSTAT, a device that performs multiple clinical chemistry readings simultaneously, is inaccurate for glucose measurements (Larsen *et al*., 2002). Other glucometers intended for use in humans, dogs or cats also perform poorly in wild mammals, such as deer (Burdick *et al*., 2012) and prairie dogs (Higbie *et al*., 2015). In ferrets (*Mustela putorius furo*) canine, but not human glucometers, perform satisfactorily (Peritz *et al*., 2013; Summa *et al*., 2014). Unfortunately, there is no way to distinguish the accurate from inaccurate readings from the glucometer alone, and therefore a correction factor cannot be applied.

A number of possibilities may explain poor glucometer performance in seals. Firstly, we used venous whole blood, whereas glucometers are designed for capillary whole blood. However, our approach matched the sample type (i.e. both from the venous compartment) and therefore should show a closer relationship between measurements (Haeckel *et al*., 2002). We used the venous compartment to avoid multiple sampling sites on the same animal. In addition, capillary sampling in seals may not represent whole body glucose levels because capillaries often constrict during handling stress or anaesthesia.

Plasma or serum typically has 11–12% higher glucose content than whole blood at an Hct of 45% (Tonyushkina and Nichols, 2009). Glucometer measurements depend on plasma proteins and number of cells present. The One Touch^®^ Ultra contains a 12% offset to account for the difference, and this constant may not be appropriate for seals (Castellini and Castellini, 1989). For human blood, the One Touch^®^ Ultra is accurate for Hct between 30 and 55% and high Hct can reduce the glucose measurement. Grey seal Hct is 40–52% (Hall, 1998; Noren *et al*., 2008) and therefore should be within the device tolerance. However, Hct in seals increases rapidly to over 60% during breath holding or diving (Castellini *et al*., 1996), during epinephrine induced splenic contraction (Hurford *et al*., 1996; Cabanac *et al*., 1997), and changes with age (Hall, 1998; Noren *et al*., 2008). Variation in breathing rate and splenic contraction during handling stress or anaesthesia in grey seals may produce large Hct fluctuations, and alter pH and oxygenation, which can affect glucometer readings (Tang *et al*., 2001).

Glucometers are calibrated for erythrocyte settling rate, which is 1–4 mm h^−1^ in grey seal pups (Hall, 1998) compared with 12–23 mm h^−1^ in humans (Wetteland *et al*., 1996). Seal haemoglobin (Hb) concentration is also higher (Noren *et al*., 2008). Differences in any or all of these conditions in seal blood compared with normal human blood could prevent correlation between the glucometer readings and laboratory analysis. While it may be possible to calibrate the glucometer by taking Hct, settling rate and/or water content into consideration, because these measurements can be performed in the field in some circumstances, the need to obtain plasma samples for calibration would negate the value of the glucometer for minimizing sample volume, and another POC or an Hct centrifuge would be required in the field. Glucometers that are intended for use in companion animals (Weiss and Reusch, 2000; Zini *et al*., 2009), which have more similar Hct and settling rates to seals (Stephens, 1938), or those that measure Hct simultaneously (e.g. Rao *et al*., 2005), may perform better for species with high and variable Hct. Interestingly, accounting for Hct, red blood cell count, Hb, total protein levels or creatinine did not improve the glucometer estimates in ferret venous blood (Summa *et al*., 2014), suggesting that additional factors affect device performance.

We saw a weak correlation between plasma and glucometer readings in pups only late in the suckling period, which suggests that this age/developmental category has the lowest circulating levels of interfering substances. This is surprising given that suckling pups are likely to have the highest levels of circulating fats (Schweigert, 1993), which can interfere with glucometer performance at high levels (Heinemann, 2010; Lindholm and Altimiras, 2016). Reported triglyceride levels in grey seals should be well within the tolerance of the device we used (<34.2 mM: Heinemann, 2010), whereas cholesterol levels in postweaned pups could approach levels that cause interference (<18.1 mM: Schweigert, 1993; Mazzarro *et al*., 2003).

Glucometers and test strips are sensitive to temperature and moisture changes (King *et al*., 1995; Tonyushkina and Nichols, 2009). Our device and strips were kept dry until use, field conditions fell within the 10–90% humidity range recommended and we avoided sampling during rain. Inaccuracy may be introduced by other field conditions, such as contact of needle with fur, skin and fat, or presence of dirt and sea spray.

### Stability of blood glucose

Glucose concentration falls by 5–7% per hour in human blood when serum or plasma is in contact with erythrocytes (Tonyushkina and Nichols, 2009), dropping by 25% within 4 h and 75% within 24 h of sampling (Lin *et al*., 1976; Chan *et al*., 1989; Fobker, 2014). Here, chilling avoided rapid glycolysis, which is an advantage in field situations in which substantial delays between sampling and processing can occur.

We saw a small but significant increase in glucose with time elapsed between sampling and removal of the plasma. Glucose production by the cellular portion is unlikely (Bennett and Burchell, 2013; Jun *et al*., 2012). Hb, a leading cause of interference in clinical chemistry assays (Grafmeyer *et al*., 1995; Lippi *et al*., 2008), was removed by deproteinization. However, our assay detects hydrogen peroxide (H_2_O_2_), which can be produced by deproteinization of Hb (Galleman and Eyer, 1990). H_2_O_2_ may then interfere in proportion to haemolysis in the original sample. EDTA and cold storage produce greater haemolysis (Muro *et al*., 1998; Mafuvadze and Erlwanger, 2007; Walencik and Witseka, 2007). Sodium fluoride and sodium citrate in Vacutainers minimize glycolysis and avoid the need to chill samples. However, these inhibitors take up to 4 h to take effect (Cavalier *et al*., 2008; Mikesh and Bruns, 2008; Gambino *et al*., 2009) or interfere with enzymes used in glucose determination (Waring *et al*., 2007). Although not an issue in large animals such as seals, draw volume from small mammals and birds can be limited, which may lead to under-dilution of stabilizers. Based on the small but statistically significant increase in blood glucose with time on the clot, we recommend plasma be removed from the cellular fraction as soon as possible, irrespective of chilling, and that direct contact with ice packs is avoided.

### Impact of method on conclusions regarding blood glucose levels in seals

Our plasma glucose measurements show no change in grey seal pups, either during suckling or after weaning, similar to Hall (1998) and Schweigert (1993). Circulating glucose in females fell from early to late in lactation. Group differences were not a result of differences in time taken to process the samples. Fasting, lactating adult female elephant seals show lower glucose levels (6.96–8.16 mM) than those reported here, and either no change or an increase in glucose levels during lactation (Houser *et al*., 2007; Fowler *et al*., 2008). Typically, elevated EGP is required in other mammals to support milk production (Mohammad *et al*., 2009). Glucose may thus be higher in grey seals earlier in lactation to support high milk output (Mellish *et al*., 1999; Mellish and Iverson, 2000). The reduction in circulating glucose later in lactation may reflect decreased EGP to protect protein reserves. In contrast to the plasma values, the glucometer suggested glucose was stable in mothers during lactation, and fell from suckling to fasting in pups. Conclusions based on the glucometer would thus be inappropriate for health or physiological assessment.

Glucose levels show diurnal fluctuations in humans (Sennels *et al*., 2015), but we saw no time of day effect. Since diurnal glucose changes were too small to be detected it appears less critical to standardize time of day, which can be difficult in field conditions, compared with the need to process samples rapidly.

## Conclusion

In other species with high glucose and Hct (e.g. penguins: *Pygoscelis* sp., Ibaňez *et al*., 2015), glucometer readings have been assumed to be usable based on similarities to values seen in prior studies (e.g. Campos Medeiros *et al*., 2011). Although the glucometer readings here were similar to glucose concentrations found elsewhere in grey seals (Schweigert, 1993; Hall, 1998; Bennett *et al*., 2013), they differ markedly from the plasma glucose results in matched samples. The discrepancy between the two methods produces different conclusions about typical changes that occur in mothers and pups during suckling and fasting. In species of conservation concern (e.g. Rea *et al*., 1998; Reif *et al*., 2004) such differences could result in use of inappropriate management criteria. Our data highlight that assumptions about glucometer accuracy based on similarities in the range of values obtained are not sufficient, and underscore the need for standardization and care in blood sample collection and handling to minimize artefactual effects on laboratory glucose measurements. A glucometer for use in species or situations when high and fluctuating Hct and lipids are expected, and variable temperature and humidity are likely to occur, would present significant advantages for use in wildlife medicine, conservation and management, and basic research.
